# Case Report: ACTH- and CRH-secreting pheochromocytoma as a very rare cause of ectopic Cushing syndrome

**DOI:** 10.3389/fendo.2025.1580876

**Published:** 2025-11-17

**Authors:** Barbara Bromińska, Nadia Sawicka-Gutaj, Ewelina Szczepanek-Parulska, Małgorzata Lange-Ratajczak, Tomasz Wierzbicki, Małgorzata Janicka-Jedyńska, Rafał Czepczyński, Marek Ruchała

**Affiliations:** 1 Department of Endocrinology, Metabolism and Internal Medicine, Poznan University of Medical Sciences, Poznan, Poland; 2 Clinical Department of General, Gastroenterological and Endocrine Surgery, Poznan University of Medical Sciences, Poznan, Poland; 3 Department of Clinical Pathomorphology, Poznan University of Medical Sciences, Poznan, Poland

**Keywords:** pheochromocytoma, adrenal gland, ectopic hypercortisolemia, Cushing syndrome, catecholamines

## Abstract

Ectopic adrenocorticotropic hormone (ACTH) syndrome (EAS) represents a rare clinical entity characterized by severe hypercortisolemia. Pheochromocytoma, a neuroendocrine neoplasm originating from chromaffin cells within the adrenal medulla, is itself an uncommon diagnosis. The phenomenon of ectopic hormone production by pheochromocytomas is exceedingly rare, occurring in less than 1% of cases. In most instances, hypercortisolemia associated with pheochromocytoma is attributed to aberrant ACTH secretion. This report presents an exceptional case of EAS caused by a pheochromocytoma exhibiting co-expression of both ACTH and corticotropin-releasing hormone (CRH).

## Introduction

Ectopic adrenocorticotropic hormone (ACTH) syndrome (EAS) is a very rare condition, characterized by symptoms of intense exposure of the body to hypercortisolemia. Cushing syndrome is an uncommon disease with an incidence of approximately 0.7–2.4 cases per million per year. In most Cushing syndrome patients, excessive endogenous cortisol production is ACTH-dependent (80%–85%) (1). Among those, pituitary adenomas are the most prevalent cause of Cushing syndrome, while in approximately 9%–18%, the source of unregulated ACTH secretion is of extra-pituitary origin (2, 3). Neuroendocrine tumors presenting as an EAS can be located in various organs. They differ in histological type and aggressiveness. The localization of the source of ACTH secretion is challenging (4). While clinical presentation is related to tumor characteristics, severe EAS should be treated as an endocrine emergency (5). Because of the rarity of the condition, there are no evidence-based guidelines concerning EAS.

The detailed molecular mechanisms behind EAS is still not completely understood (6). Some EAS-inducing tumors have shown involvement of epigenetic mechanisms, especially the hypomethylation of the proopiomelanocortin (POMC) promoter (7, 8). Additionally, the specific expression of transcription factors that promote ACTH production in these tumors has been proposed (9).

Pheochromocytoma is a rare tumor derived from the chromaffin cells of the adrenal medulla. The annual incidence is six to seven cases per million people per year. In patients with incidentally detected adrenal masses, 0.6% to 4.2% is identified as pheochromocytoma. Such a tumor typically secretes catecholamines. As a result, the most characteristic symptoms include headaches, sweating, hypertension, palpitations, and pallor (10). Pheochromocytoma can also produce other hormones such as interleukins, calcitonin, ACTH, or corticotropin-releasing hormone (CRH). Ectopic hormone production from pheochromocytoma is an extremely rare occurrence, estimated to be found in less than 1% of all cases. Ectopic ACTH or CRH changes the typical presentation of pheochromocytoma. Contrary to pheochromocytomas in general, patients are predominantly women. They exhibit a hypercortisolemic phenotype rather than typical symptoms of pheochromocytoma (11, 12).

Delayed diagnosis of pheochromocytoma may have life-threatening consequences, but the timely recognition of the tumor co-secreting catecholamines and cortisol is crucial.

The importance of the diagnosis is underlined by the historically reported survival rate of 43%. Nowadays, risk of complications and death is lower, but still significant. The last update of diagnostic criteria for pheochromocytoma-related ectopic hypercortisolemia was made in 1995. It included both pre-surgical, clinical, biochemical, and radiological confirmation of pheochromocytoma and ACTH-dependent hypercortisolemia. Post-surgical ACTH normalization and resolution of both signs and symptoms is significant (13). However, with the evolution of diagnostic techniques and shifts in epidemiology over time, it remains uncertain whether these criteria still accurately reflect most contemporary cases.

This report aims to delve deeper into the topic of ectopic hypercortisolemia originating from ACTH- and CRH-secreting pheochromocytoma, illustrated through the example of our patient. According to our knowledge, this is the fourth case in the world (10, 14).

## Case report

### Presentation

**Figure 1:**
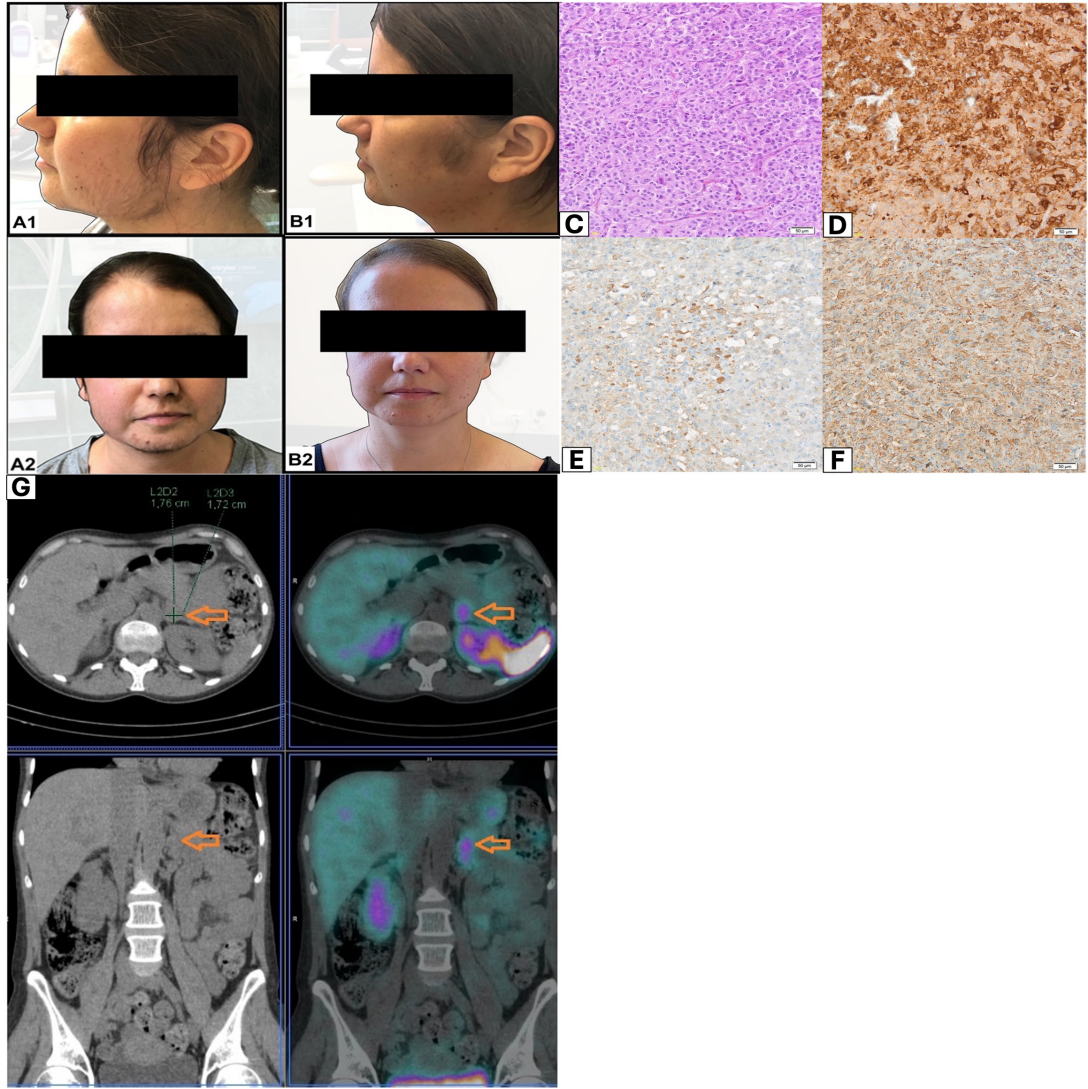
**(A, B)** Patient appearance before and after adrenalectomy. **(C)** Histopathological evaluation revealed pheochromocytoma of the adrenal gland. H +E method; magnification 20×. **(D)** In the immunohistochemical assessment: C-CRH-positive cells; magnification 10×. **(E)** In the immunohistochemical assessment: ACTH-positive cells; magnification 20×. **(F)** In the immunohistochemical assessment: chromogranin A-positive cells; magnification 20×. **(G)** Whole-body scan and SPECT/CT of the abdomen were performed 3 h after 99mTc-HYNIC-TOC administration. Increased uptake of the tracer was localized in tumor of the left adrenal gland (orange arrows).

A 36-year-old woman presented with muscle weakness and tremor, easy bruising, hirsutism, androgenic alopecia, facial acne, secondary amenorrhea, and deterioration of visual acuity. The patient lost 17 kg within 5 months (her body mass index on admission was 19.8 kg/m^2^; **Figures 1A, B**). In addition, ulceration around the tongue and oral mucosa was observed. The patient has been recently diagnosed with diabetes mellitus and hypertension. On admission, she had profound hypokalemia. While cortisol rhythm was stiff, ACTH was markedly elevated. Cortisol in 24-h urine collection was 25 times higher than the upper normal limit. Levels of catecholamines in 24-h urine collection were normal. Additionally, dehydroepiandrosterone-sulfate (DHEA-s), testosterone, liver enzymes, cholesterol, and natrium levels were high. The patient was diagnosed with hypogonadotropic hypogonadism: follicle-stimulating hormone (FSH) <0.3 mIU/mL, luteinizing hormone (LH) < 0.3 mIU/mL, and estradiol 32 pg/mL; and secondary hypothyroidism: thyroid-stimulating hormone (TSH) 0.07 μU/mL (norm 0.27–4.2), free triiodothyronine (ft3) 3.52 pmol/L (norm 3.9-6.7), and free thyroxine (ft4) 20 pmol/L (norm 11.5–21.0 pmol/L). Functional tests with dexamethasone and CRH were performed.

Pre-operative results of laboratory tests are shown in **Table 1**.

**Table 1:** Biochemical results before and after surgery.

Parameter	Before	After
ACTH [pg/L] [7.2–63.3]	338	24.6
Cortisol [nmol/L]		
8 a.m. [166–507]	>1,750	427
6 p.m. [74–291]	>1,750	215
11 p.m. [74–291]	1,449	106
Cortisol in 24-h urine collection excretion [nmol/L] [12–486]	12,634	43
Potassium in 24-h urine collection–excretion [mmol/24 h] [25–125]	130	78
Sodium in 24-h urine collection–excretion [mmol/24 h] [40–220]	473	125
Metanephrine in 24-h urine collection–excretion [μg/24 h] [<350]	223 131	22 24
Normetanephrine in 24-h urine collection–excretion [μg/24 h] [<600.0]	444 321	168 183
Chromogranin A [ng/mL] [19–100]	480.96	42.62
Testosterone [nmol/L] [0.2–0.9]	5.87	<0.1
DHEA-S [μg/dL] [61–337]	599	38.1
Androstenedione [ng/mL] [0.4–3.4]	>10	0.65
Renin [μIU/mL] [2.8–39.9)	2.2	240.8
Aldosterone [ng/dL] [1.76–23.3]	3.49	25.0
Sodium [mmol/L] [136–145]	146	138
Potassium [mmol/L] [3.5–5.1]	2.35	4.29
Cortisol in DSST [nmol/L]	1,314	–
Cortisol in HDDST [nmol/L]	1,613	–
Cortisol in CRH test—before the operation [nmol/L]	0′ 1,807 15′ 1,865 30′ 1,942 60′ 1,908 120′ 1,905	

ACTH, adrenocorticotropic hormone; DHEA-S, dehydroepiandrosterone sulfate; LDDST, low dose dexamethasone suppression test; HDDST, high-dose dexamethasone suppression test; CRH, corticotropin-releasing hormone; PPI, proton pump inhibitors.

Because of suspicion of ectopic Cushing syndrome, extensive imaging diagnostics was conducted.

### Diagnosis

Adrenal computed tomography (CT) scan showed a lipid-poor adenoma in the left adrenal gland (diameter of 17 mm; initially density of 34 HU; contrast washout index of 62%). Magnetic resonance imaging (MRI) of the pituitary gland did not visualize any significant abnormalities. Whole-body scan and single-photon emission computed tomography (SPECT) (**Figure 1G**) of the abdomen were performed 3 h after 99mTc-HYNIC-TOC administration. Scans revealed increased uptake of the administered tracer in a lipid-poor adenoma of the left adrenal gland.

### Management

The patient required intensive insulin therapy. Potassium levels were corrected with intravenous and oral supplementation. Metyrapone was implemented (maximal dose, 1,500 mg), which resulted in measurable values of cortisol in the blood serum (<1,000 μg/dL), improvement in glycemic control, and correction of electrolyte disturbances. Since blood pressure stabilized following the start of therapy and 24-h urinary catecholamine levels remained within normal limits, alpha-blockers were not implemented. After preparations, the patient underwent laparoscopic left-sided adrenalectomy. The operation proceeded without complications.

Histopathological evaluation revealed a pheochromocytoma. Five points were given in the PASS scale: cellular monotony (score = 2), diffuse growth (score = 2), and vascular invasion (score = 1). Histopathological evaluation comprised standard hematoxylin and eosin (H&E) staining, supplemented by immunohistochemical (IHC) analysis targeting chromogranin A (CgA), synaptophysin, CRH, and ACTH. ACTH detection was performed using a rabbit polyclonal antibody (Roche, Basel, Switzerland), while CRH was identified using a rabbit polyclonal CRH/CRF antibody (Proteintech, Rosemont, USA; Cat# 10944-1-AP). In the IHC assessment: ACTH (+), CRH (+), synaptophysin (+), chromogranin (+), and Ki-67 2% (**Figures 1C–F**).

After the surgery, during the CRH test, an adequate response of the pituitary corticotropic axis was obtained. In addition, insulin therapy was discontinued, and antihypertensive therapy was reduced. Three months later, further follow-up examinations were performed. The patient was asymptomatic and did not require hormonal replacement therapy. The circadian rhythm of cortisol was restored, and there was a correct response in the synthetic ACTH stimulation test. Luteinizing hormone and follicle-stimulating hormone levels normalized. Thyroid-stimulating hormone axis returned to normal (TSH, 0.72 μU/mL; ft3, 5.43 pmol/L; and ft4 15.7, pmol/L). There was no need for oral medication. Only supplementation of vitamin D was continued. *RET* gene examination did not reveal mutations. Abdominal CT scan did not demonstrate signs of recurrence in the left adrenal bed.

Post-operative results of laboratory tests are shown in **Table 1**.

## Discussion

Pheochromocytoma/paraganglioma accounted for about 5% of ectopic ACTH/CRH-secreting tumors. They release mainly mature, bioactive ACTH. Exceptionally, tumors produce only CRH (15). Hereby, we present a patient with pheochromocytoma secreting both ACTH and CRH, which is extremely rare.

The clinical picture of ectopic Cushing syndrome depends on tumor characteristics, but it is often associated with severe hypercortisolism. This, in turn, is responsible for a wide variety of complications and comorbidities (16). Mortality rates are increased (17, 18); thus, the primary goal in case of severe ectopic Cushing syndrome is the rapid control of hypercortisolemia (19). The clinical picture becomes even more challenging when overlapping symptoms of pheochromocytoma and Cushing syndrome must be considered. Moreover, co-expression of ACTH and CRH can significantly modify the course of the disease.

Typically, the median period from the first symptoms to diagnosis is 4 months. In contrast, for other ectopic and pituitary Cushing syndrome, the time to establish a diagnosis is significantly longer—14 and 38 months, respectively (20). The early presentation in ACTH/CRH-producing pheochromocytomas might be attributed to synergistic effects of glucocorticoids and catecholamines. The former alters phenylethanolamine-N-methyltransferase (PNMT) function, which is the enzyme transforming adrenal norepinephrine to epinephrine. The latter impacts steroidogenesis, probably through paracrine mechanisms (21). Moreover, co-expression of ACTH and CRH has the potential to markedly deteriorate the disease’s course. Nakhjavani et al. analyzed a series of 75 ectopic Cushing syndrome associated with CRH secretion. They showed that tumors producing CRH are associated with higher serum cortisol levels than those releasing ACTH. Diabetes and hypokalemia were very common. Prognosis, despite treatment, was worse, when compared with other types of hypercortisolemia (22).

To date, only three cases of pheochromocytoma co-secreting ACTH and CRH have been documented. Each involved female patients presenting with clinical features of acute hypercortisolemia and elevated levels of both urinary cortisol and metanephrines (10, 14).

Clinically, our patient exhibited signs of hypercortisolemia without evidence of metanephrine excess. This aligns with findings by Elliot et al. (81% of cases showed Cushingoid features) (10) and Kishlyansky et al. (82%). Blood pressure was well-controlled after the introduction of anti-hypertensive drugs and steroidogenesis inhibitors. While catecholamine levels were not elevated in the 24-h urine collection, cortisol secretion was 25-fold higher than the upper limit of norm. In contrast, in a study by Kishlyansky et al. (23), urinary metanephrines in ACTH/CRH-secreting pheochromocytoma/paraganglioma were increased at least three times above normal in 74% of the patients, while the same is true for normetanephrine in 52% of the patients (23). Accordingly, we have considered the possibility of “silent” pheochromocytoma (24). While preoperative alpha-blockade is widely accepted for hypertensive pheochromocytoma, its role in being biochemically “silent” is less clear. Managing these cases requires an individualized approach, balancing the risk of intraoperative hypertension with potential postoperative hypotension. Consequently, alpha-blockers were not introduced (25).

Our patient initially presented with newly diagnosed diabetes mellitus, hypertension, myopathy, and hypokalemia. In recent reviews concerning ACTH-producing pheochromocytoma by Elliott et al. (10) and Kishlyansky et al., the rates of concomitant disorders were as follows: hypertension: 93% vs. 86%; hyperglycemia: 54% vs. 62%; and psychiatric symptoms: 27% vs. 25%. Marked hypokalemia was common and reported in 83% of the cases by Elliott, with median potassium serum levels of approximately 2.7 mmol/L. Our patient required insulinotherapy, as in 94% of cases reported by Kishlyansky et al., and the incidence of newly diagnosed diabetes was 63% (23).

After extensive imaging diagnostics, our patient underwent laparoscopic left-sided adrenalectomy. Histopathological evaluation revealed a pheochromocytoma producing both ACTH and CRH. Diagnosis was made based on clinical and pathophysiological findings. ACTH and CRH expression in removed tumor characterized as pheochromocytoma was confirmed by immunohistochemistry.

Recent meta-analysis described 99 patients with pheochromocytomas causing ectopic Cushing syndrome. Expression of exclusive ACTH was found in majority of tumors, 93%, while CRH was present in 5% of cases. Co-expression of CRH and ACTH, as in our case, was detected only in 2 of 84 immunohistochemically assessed tumors (10). In the remaining case described by O’Brien et al., the pheochromocytoma was found to express not only ACTH and CRH but also vasopressin (26).

However, the incidence of CRH-ectopic Cushing syndrome may be underestimated. When the non-pituitary tumor gains the ability to secrete CRH, it stimulates the pituitary gland to produce excessive ACTH. This leads to a possible diagnostic pitfall by mimicking Cushing disease, owing to false-positive results of bilateral inferior petrosal sinus sampling (19).

Additionally, diagnostic criteria for ectopic Cushing syndrome differ across published case series. The diagnosis was based on immunostaining for CRH, plasma CRH measurement, *in vitro* tumor studies, or gradient of CRH concentration (22).

## Conclusions

We presented a rare case of ectopic Cushing syndrome due to ACTH- and CRH-secreting pheochromocytoma. Ectopic Cushing syndrome is characterized by serious complications and significant morbidity associated with hypercortisolism and tumor spread. In case of severe hypercortisolism, the diagnostic process should not be prolonged, and the main goal is to achieve cortisol secretion control. Although ACTH/CRH-secreting pheochromocytoma is extremely rare, timely recognition of this tumor is crucial. Ectopic simultaneous ACTH and CRH production can change the typical presentation of pheochromocytoma, impeding a diagnostic process. While complications are severe, identification and proper management leads to a cure rate of 92%, further underlying the need for prompt diagnosis.

## Data Availability

The original contributions presented in the study are included in the article/supplementary material. Further inquiries can be directed to the corresponding author.
